# Chemically Cross-Linked Graphene Oxide as a Selective Layer on Electrospun Polyvinyl Alcohol Nanofiber Membrane for Nanofiltration Application

**DOI:** 10.3390/nano11112867

**Published:** 2021-10-27

**Authors:** Myoung Jun Park, Grace M. Nisola, Dong Han Seo, Chen Wang, Sherub Phuntsho, Youngwoo Choo, Wook-Jin Chung, Ho Kyong Shon

**Affiliations:** 1 Centre for Technology in Water and Wastewater (CTWW), School of Civil and Environmental Engineering, University of Technology Sydney (UTS), P.O. Box 123, 15 Broadway, NSW 2007, Australia; MichaelDongHan.Seo@uts.edu.au (D.H.S.); Chen.Wang-7@student.uts.edu.au (C.W.); Sherub.Phuntsho@uts.edu.au (S.P.); Youngwoo.Choo@uts.edu.au (Y.C.); 2 Environmental Waste Recycle Institute (EWRI), Department of Energy Science and Technology (DEST), Myongji University, Myongji-ro 116, Cheoin-gu, Yongin-si, Gyeonggi-do 17058, Korea; grace.nisola@gmail.com (G.M.N.); wjc0828@gmail.com (W.-J.C.)

**Keywords:** graphene oxide, nanofiber, electrospinning, polyvinyl alcohol, cross-linking, nanofiltration

## Abstract

Graphene oxide (GO) nanosheets were utilized as a selective layer on a highly porous polyvinyl alcohol (PVA) nanofiber support via a pressure-assisted self-assembly technique to synthesize composite nanofiltration membranes. The GO layer was rendered stable by cross-linking the nanosheets (GO-to-GO) and by linking them onto the support surface (GO-to-PVA) using glutaraldehyde (GA). The amounts of GO and GA deposited on the PVA substrate were varied to determine the optimum nanofiltration membrane both in terms of water flux and salt rejection performances. The successful GA cross-linking of GO interlayers and GO-PVA via acetalization was confirmed by FTIR and XPS analyses, which corroborated with other characterization results from contact angle and zeta potential measurements. Morphologies of the most effective membrane (CGOPVA-50) featured a defect-free GA cross-linked GO layer with a thickness of ~67 nm. The best solute rejections of the CGOPVA-50 membrane were 91.01% for Na_2_SO_4_ (20 mM), 98.12% for Eosin Y (10 mg/L), 76.92% for Methylene blue (10 mg/L), and 49.62% for NaCl (20 mM). These findings may provide one of the promising approaches in synthesizing mechanically stable GO-based thin-film composite membranes that are effective for solute separation via nanofiltration.

## 1. Introduction

In recent years, graphite-based materials such as graphene, graphene oxide (GO), and reduced graphene oxide (rGO) have gained significant attention as additives or components in membrane fabrication of water purification membranes [1,2,3,4,5,6,7,8]. These materials feature salient characteristics such as ease of fabrication, chemical amenability to functionalization, and high mechanical strength [9,10,11]. Among the graphite derivatives, a single-layer GO is a two-dimensional (2D) atom-thick material that has demonstrated great potential for water separation membranes [12,13,14,15,16]. In particular, GO nanosheets contain oxygenous moieties such as carboxyl (–COOH), epoxy (–O–), and hydroxyl (–OH) groups on surfaces and edges that can be exploited in fabricating functional nanocomposite membranes with high chemical stability, high hydrophilicity, and excellent antifouling and antibacterial properties [2,4,17,18,19].

Previously, graphene and GO nanosheets have been incorporated in composite membranes using various polymeric support materials [20,21,22]. Porous graphene membranes have been developed for specialized membrane-based separation applications. However, despite the simulations and experimental efforts, technical difficulties in creating such membranes for real-world water separation applications have yet to be resolved [23]. For example, it is still impractical to prepare a large-area graphene membrane, and it remains challenging to generate high density of nanoporous GO sheets with controllable and relatively uniform GO sheets sizes.

To address these predicaments, an alternative approach is to use the currently available GO nanosheets by stacking them on a polymeric support to create a selective layer and produce composite water filtration membranes [1,7,24,25]. The GOs can serve as effective physical barriers to reject and retain solutes at the feed side [26]. Moreover, as 2D materials, the stacked GOs can create pores between sheets as channels for water transport [11]. Multi-layer GO stacking could refine the membrane selectivity by creating tortuous paths for the water and solute, effectively separating the two components. However, stacked GOs are very hydrophilic, which renders them susceptible to damage during membrane operation as the GO sheets can easily detach and re-disperse in water [25,27,28,29,30]. Thus, the GO-selective layer requires reinforcements to ensure its mechanical stability for long-term application. For instance, the stacking of GO nanosheets via π–π interactions has been effectively utilized for the preparation of GO-selective layers [30]. On the other hand, chemical reinforcements using different cross-linking agents such as diamines [31,32,33], polydopamine [28,34,35], urea [25], polyelectrolytes [1,27,36], tannic acid [37], and glutaraldehyde (GA) [38,39,40] have also been used to enhance the chemical and physical stabilities of GO nanosheets. For example, Jia and coworkers used p-phenylenediamine (PPD), and Peng and coworkers used ethylene diamine to obtain mechanically robust GO membranes [32,33]. Zhan and coworkers successfully fabricated a durable GO composite nanofibrous membrane with bioinspired polydopamine for efficient separation of anionic dyes [35]. A polyelectrolyte complex with a GO composite was successfully constructed as an efficient barrier for water desalination [27]. 

However, most of these approaches have focused on stability among GO sheets but have not ensured the strong interaction between the GO layer and its support (i.e., microfiltration or ultrafiltration substrates). Without interlayer stability, the GO layer can still detach during operation, which could affect its long-term performance. Although π–π interactions have been used to fixate a GO layer on its support, this method is only effective if the polymer substrates also contain aromatic π systems [30,33]. Thus, this approach may not be applicable to various types of supports. Alternatively, the same outcome can be achieved via chemical cross-linking. The entire GO layer can be covalently attached on any type of polymeric substrate as long as the selected cross-linker is reactive to both layers. With an intact membrane, GO sloughing can be avoided, for example, in a cross-flow filtration application.

Among the different types of cross-linkers, GA has been used for the development of physically and chemically stable GO composite membranes intercalated with polyvinyl alcohol (PVA), mostly used in pervaporation [38,39,40]. For GO composite nanofiltration (NF) membranes, GA is likewise an ideal cross-linker as it can form acetal bonds with both GO and PVA, as both materials are rich in GA-reactive –OH groups [40,41,42,43]. Covalent linkages between GO and PVA layers via GA cross-linking could afford a robust GO/PVA composite NF that is highly stable in water, which is favorable during operation. So far, the construction of a GA-cross-linked GO layer that is covalently attached on a porous electrospun PVA support has yet to be materialized. Moreover, the use of electrospun PVA is widely unexplored as a membrane support layer for the production of thin-film-composite NF membranes. The highly porous PVA nanofiber support can significantly reduce the membrane resistance and enhance water permeation in the presence of a GO-selective layer [35,39,44,45,46].

**Figure 1:**
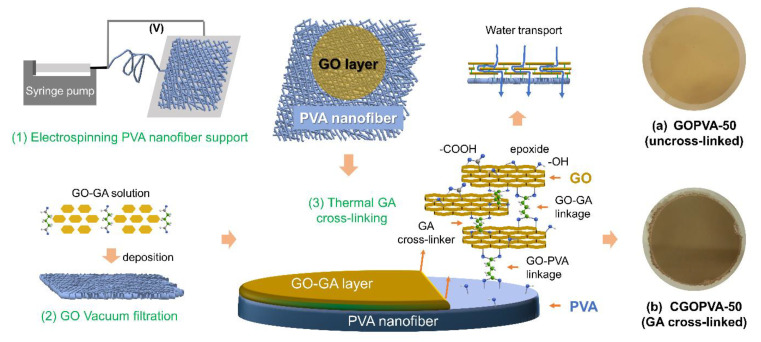
Schematic illustration for the preparation of the cross-linked GO-PVA composite membrane (CGOPVA-50) by GA cross-linker.

Thus, in this study, a simple approach of creating a stable and highly selective GO layer on a porous electrospun PVA nanofiber as support was first presented via chemical cross-linking with GA. Novel GO thin-film composite NF membranes were developed by depositing a thin layer of GO with a GA cross-linker on top of the PVA support through a simple pressure-assisted self-assembly technique (Figure 1). In the presence of mild heat and an acid catalyst, GA linkages between the GO sheets, as well as between the GO layer and PVA support, were formed to enhance the overall mechanical stability of the composite membranes. Meanwhile, the pore size or the interlayer space between GOs was controlled by varying the deposition amount of GO on the PVA support. A series of characterization techniques were carried out to examine the properties of the developed composite membranes. To highlight the benefits of GA cross-linking for the GOPVA composite membranes, control samples without GA were also characterized and evaluated for nanofiltration operation in terms of pure water permeability, flux, and salt rejection efficiencies using different types of feed solutions.

## 2. Experimental

### 2.1. Reagents

Polyvinyl alcohol (PVA, Mw = 85,000~124,000, 87~89% hydrolyzed), surfactant Triton-X 100 (laboratory grade), and cross-linker glutaraldehyde (GA) (25% aqueous solution) were purchased from Sigma-Aldrich Inc. (St. Louis, MO, USA). Catalyst hydrochloric acid (HCl, 32%) was purchased from Chem-supply Pty Ltd. (Bedford St. Gillman, SA, Australia). Single-layer GO nanosheets from ACS (Advanced Chemicals Supplier, Walnut St. Pasadena, CA, USA) prepared by the modified Hummers’ method were used in this study without further purification. Acetone (99.8%) and ethanol (96%) were purchased from Chem-supply Pty Ltd. and Ajax Finechem Pty Ltd. (Auburn St. Wollongong, NSW, Australia), respectively. Methylene blue (MB) (laboratory regent) from Ajax Finechem Ltd. and eosin Y (EY, Dye content ~99%) from Sigma-Aldrich were used as dye solutions for rejection performance evaluation. Sodium chloride (NaCl) (≥99.7%, analytical reagent) from Chem-Supply Pty Ltd. was used for salt rejection experiment. Divalent metal salts such as sodium sulfate (Na_2_SO_4_) (99.5%, anhydrous), magnesium sulfate heptahydrate (MgSO_4_∙7H_2_O) (≥98%, ACS reagent) and magnesium chloride (MgCl_2_) (≥98.0%) from Sigma-Aldrich were also used for salt rejection tests. All chemicals were used without further purification.

### 2.2. Preparation of Electrospun PVA Nanofiber Membrane

A highly porous PVA nanofiber support was prepared via the electrospinning technique (Figure 1). The polymer solution was prepared by dissolving 10 wt% of PVA powder in deionized (DI) water for 3 h at 90 °C with stirring (270 rpm). The PVA solution was further stirred for another 21 h at room temperature. To decrease the surface tension of the polymer solution, Triton X-100 was added as a surfactant to the solution with a concentration of 0.6 v/w%, and then the dope solution was further stirred for another 15 min prior to electrospinning.

The dope solution was transferred into a syringe, which was mounted on a syringe pump for constant fluid delivery through a G21 (ID 0.51 mm) metal nozzle. Overall, 8 mL of solution was supplied at a flow rate of 0.8 mL/h and a voltage of 28 kV was applied in the electrospinning unit to create the nanofibers, which were collected on a rotating drum (100 rpm) covered with aluminum foil. The distance between the nozzle and drum collector was fixed at 100 mm. During the electrospinning, the temperature was maintained at 23~25 °C, whereas the humidity was controlled at 35~55% inside the electrospinning chamber. The collected PVA nanofiber sheet was sandwiched between flat metal plates and treated overnight at 60 °C in an oven to remove the residual solvent (water) and obtain the compressed nanofiber sheet. 

As PVA is water-soluble, the nanofiber membrane was cross-linked with GA to render it stable in water. The nanofibers were soaked in GA cross-linking solution (0.05 M of GA in acetone with 0.02 M of HCl as catalyst) for 4 h at room temperature. The cross-linked PVA (CPVA) nanofiber membrane was subsequently washed with DI water several times until the washing solution reached neutral pH (6–7). The washed CPVA membrane was then dried at room temperature.

### 2.3. Deposition of GO on PVA Nanofiber Membrane via Vacuum Filtration

The GO-GA solution was prepared by dispersing 5 mg of GO powder in 1 L of DI water via ultrasonication (230 W) for 90 min. Afterward, GA (0.05 M) and HCl (0.05 M) were added in the GO (5 mg/L) and the mixture was homogenized for 30 min with magnetic stirring at room temperature. The GO-GA solutions were then deposited on PVA nanofibers (effective area = 10.18 cm^2^) via vacuum filtration. The filtration volumes of GO-GA solutions were varied from 10 to 50 mL to achieve different loading amounts of GO on the PVA membrane from 4.91 to 24.56 µg/cm^2^. The PVA membranes with the deposited GO-GA layer were further processed to facilitate GA cross-linking by soaking the samples in GA solution (0.05 M of GA and 0.02 M of HCl in acetone) for 12 h. The samples were labeled as CGOPVA-X, where X = 10, 17.5, 25, and 50 mL are the filtration volumes of the GO-GA solution (5 mg/L). Afterward, the samples were placed in an oven for 2 h at 80 °C to dry the membranes, as well as to enhance the GA cross-linking reaction between GO nanosheets and GO on the PVA surface. After the reaction, all membrane samples were washed with DI water until the pH of the washing solution became neutral (pH ~ 6–7). In order to remove the residual GA, the samples were further washed with ethanol and then dried at room temperature. Control membrane samples with an uncross-linked GO layer on CPVA (denoted as GOPVA) were also fabricated by filtering GO solutions (without GA) using the same filtration volumes (X) used for CGOPVA samples. The GOPVA-X membranes where immediately placed in a drying oven for 2 h at 80 °C, skipping the GA cross-linking reaction. 

### 2.4. Membrane Characterization

The morphology of a single-layer GO nanosheet was observed on a scanning probe microscope (Dimension 3100 Bruker, Billerica, MA, USA) under tapping mode. For atomic force microscopy (AFM), 100 mg/L of GO suspension was prepared and a few drops were deposited on a mica sheet. The sample was subsequently dried in an oven at 60 °C prior to characterization. Membrane morphologies were also examined using a field-emission scanning electron microscope (FE-SEM) (Zeiss Supra 55VP, Carl Zeiss AG, Oberkochen, Germany) operated at 10 kV. The average fiber diameters of the electrospun PVA membranes were measured from FE-SEM images using ImageJ^®^ software. Membrane hydrophilicities were determined by contact angle measurement using an optical tensiometer (Theta Lite 100, Biolin Scientific, Sweden) equipped with an image processing software. Mean and maximum pore sizes of the electrospun membranes were measured using a capillary flow porometer (CFP Porolux 100, Prometer NV, Belgium) under dry-up/wet-up mode. A low-surface-tension wetting agent Galwick (16 dynes/cm) was added to fill-up the membrane pores.

Porosity (ε) of the CPVA support was determined via the gravimetric method. The membranes were cut into 2 cm × 2 cm size and the weights of the dry (*m*_2_, g) and wet samples (*m*_1_, g) were measured. The density of PVA of *ρ_p_* = 1.19 g/cm^3^ was used as it was the bulk material in CPVA and its density was similar to that of GA (*ρ*, 1.06 g/cm^3^). Thus, the densities of water (*ρ_w_*, 1.00 g/cm^3^) and PVA *(ρ_p_*, 1.19 g/cm^3^) were used for the calculation of porosity (Equation (1)). The reported value was an average from five measurements for a more accurate result.

(1) $$\mathrm{Porosity}\;\left(\varepsilon \right)=\frac{\left(m_{1}-m_{2}\right)/\rho_{w}}{\left(m_{1}-m_{2}\right)/\rho_{w}+m_{2}/\rho_{p}}$$

Attenuated total reflectance–Fourier-transform infrared spectroscopy (ATR–FTIR) (IRAffinity-1, Shimadzu, Kyoto, Japan) and X-ray photoelectron spectroscopy (XPS) were also conducted to confirm the successful reaction of GA cross-linking. Additionally, membrane surface chemistry was characterized by zeta potential measurement using an Anton Paar Surpass solid-surface analysis, following the conditions reported in a previous study [47]. X-ray diffraction (XRD, Bruker D8) data were acquired with CuKα radiation (λ = 1.5418 Å) to determine the GO interlayer distances (d-spacing) of GO-PVA composite membranes in dry and wet states. Wet samples were prepared by soaking the membranes in DI water for 24 h. Before analysis, the excess water was removed on the membrane surface using a filter paper.

### 2.5. Evaluation of Membrane Performance

A laboratory-scale filtration unit was used to test the performance of the membrane samples in terms of water permeance and solute rejection. The samples were fixed in a membrane cell with an effective membrane area of 4 cm^2^. The system was operated under cross-flow mode with a 8.3 cm s^−1^ velocity and constant operating temperature of 25 °C ± 1. All samples were pre-operated for 30 min using DI water as the feed to minimize the effect of membrane compaction on the membrane performance. The permeate flux *J_w_* was calculated using Equation (2), where *V* is the volume of the permeate water (L), A is the effective membrane area (m^2^), and Δt is the time interval (h).

(2) $$J_{w}=\frac{V}{A\Delta t}$$

Different salt solutions of Na_2_SO_4_, MgSO_4_, CaCl_2_, and NaCl were used as feed for evaluating the salt rejection performance. The conductivities of the feed and permeate solutions were measured using a portable probe and meter (D-74G, Horiba scientific, Kyoto, Japan). Dye solutions of EY (10 mg/L) and MB (10 mg/L) were also used as a feed to investigate the dye rejection performance of the membranes, which were measured using a UV-Vis spectrophotometer (Shimadzu, Kyoto, Japan). Solute rejections (%*R*) were calculated using Equation (3), where $C_{f}$ and $C_{p}$ are the solute concentrations in the feed and permeate, respectively.

(3) $$R\;\left(\%\right)=100\times \left(1-\frac{C_{p}}{C_{f}}\right)$$

### 2.6. Evaluation of Membrane Stability and Reusability

The stability of the GO-selective layer on GOPVA-50 and CGOPVA-50 composite membranes was evaluated using the ultrasonication method. This test has been commonly used to evaluate the stability of GO membranes [30,47,48,49]. The membrane samples were first soaked in DI water and then ultrasonicated at 230 W from 0 to 30 min. The physical integrity of the membrane was inspected after each sonication period. The reusability of the CGOPVA-50 membrane was further evaluated by testing its performance after one day of soaking in DI water. The testing was repeated on the same membrane sample after 7 days of storage in DI water.

## 3. Results and Discussion

### 3.1. Characterization of PVA Nanofiber Support and GO-PVA Composite Membranes

**Figure 2:**
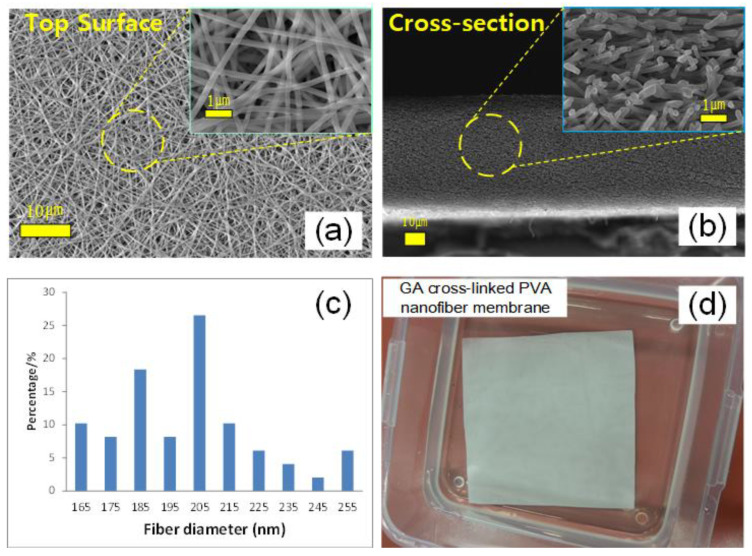
PVA nanofiber membrane prepared via electrospinning and then cross-linked via GA: SEM images of (**a**) top surface and (**b**) cross-section; (**c**) average nanofiber diameters from histogram; (**d**) water-insoluble PVA nanofiber membrane cross-linked via GA (CPVA).

The composite PVA membranes were prepared by a multi-step process (Figure 1), which first involved electrospinning of the PVA nanofiber support followed by GA cross-linking. SEM images of the CPVA nanofiber revealed its consistent and uniform morphology (Figure 2a,b). The surface image (Figure 2a) revealed bead-free nanofibers, suggesting that the electrospinning conditions were properly controlled. The fibers were well-interconnected as a result of heat-press treatment and the cross-linking of PVA fibers with GA. Meanwhile, the cross-sectional SEM image (Figure 2b) showed randomly arranged cylindrical fibers, which created the highly porous 3D matrix of the membrane support. The CPVA substrate had an average thickness of 67.52 ± 5.24 µm with a mean pore diameter of ~0.22 µm measured by CFP, which is within the rage of typical microfiltration (MF) membranes (Table 1). It can be noted that the maximum pore size measured was ~0.27 µm, which is very close to the average value. This confirms that CPVA had a narrow and uniform interstitial pore size structure, which was likely achieved due to the uniformity of the produced nanofibers with an average diameter of 203.50 ± 26.17 µm (Figure 2c and Table 1). 

The CPVA maintained its structure when submerged in water overnight (Figure 2d) as a result of a successful GA cross-linking, which indicates the stability of the membrane support in an aqueous environment [50]. A summary of the properties of CPVA is provided in Table 1. The initial filtration test revealed that the pure water permeability (PWP) of CPVA (Table 1) ~ 14,978 L m^−2^ h^−1^ bar^−1^ is remarkably higher than commercially available MF membranes fabricated via the phase separation technique [50]. The highly porous nature of CPVA ~ 84.31 ± 1.97% likely contributed to its extremely high water permeance.

**Figure 3:**
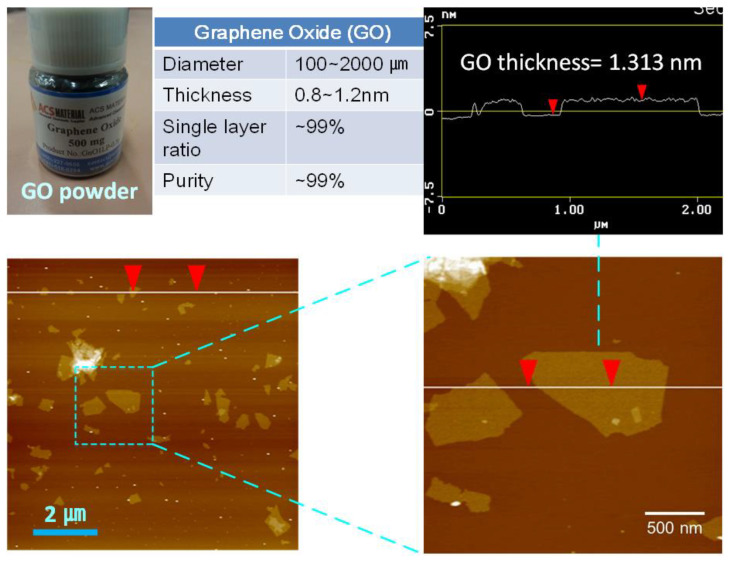
AFM images of single-layered GO nanosheets.

After the fabrication of the CPVA support, a selective layer was created on top of it by depositing the GO nanosheets through vacuum filtration. Results from AFM analysis revealed the wide lateral size distribution (100–2000 nm in lateral diameter) of the GO, which is inherent to the Hummers’ method that has little control on the size of the produced nanosheets (Figure 3). The thickness of GO was measured to be ~1.3 nm, which confirmed that it was highly pure and single layered [51]. Different types of composite membranes were prepared by varying the amount of GO deposited on the CPVA nanofiber. 

**Figure 4:**
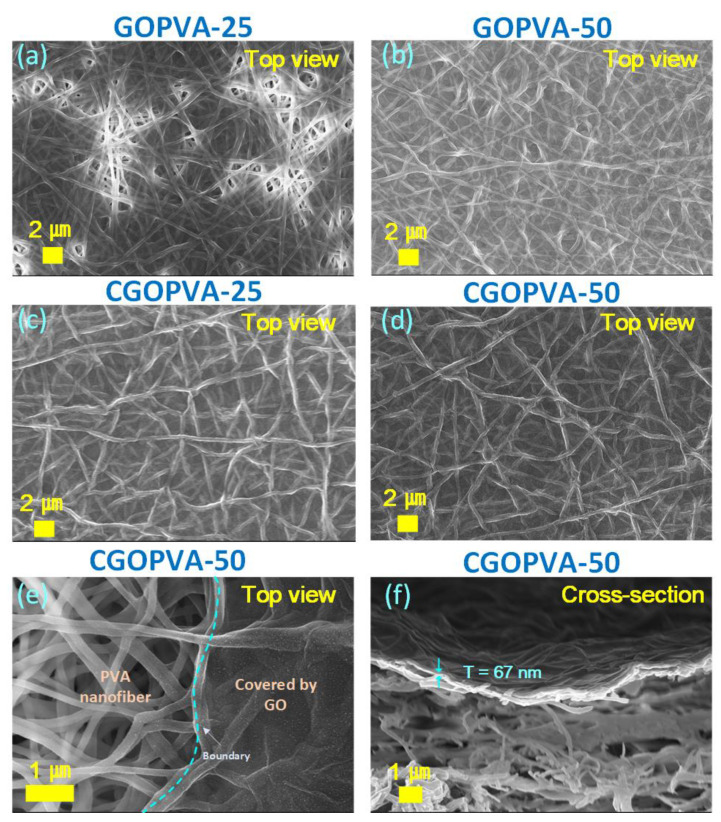
FESEM images of top view for (**a**) GOPVA-25, (**b**) GOPVA-50, (**c**) CGOPVA-25, and (**d**,**e**) CGOPVA-50 and of cross-section for (**f**) CGOPVA-50.

The surfaces of the selected composite membrane samples deposited with high GO filtration volumes were observed under FE-SEM (Figure 4). The FE-SEM image of GOPVA-25 membrane revealed that the support was not completely covered by GO, as certain regions (bright areas in the image) still had exposed open pores (Figure 4a). However, a full coverage of GO on the CPVA substrate was achieved for the GOPVA-50 membrane, suggesting that a defect-free membrane surface was produced using a filtration volume of 50 mL (Figure 4b). Both GA cross-linked samples showed a more uniform GO distribution on the surface (Figure 4c,d). The imprinted patterns of electrospun nanofibers were visible, which could be attributed to the flexibility of the stacked single-layer GO nanosheets. This type of morphology was also observed in the literature, where GO nanosheets were coated on a poly (arylene ether nitrile) nanofiber substrate [35]. The complete GO coverage in CGOPVA-25 could be attributed to the effectively coated GO-GA on the substrate, as the GO nanosheets were partially cross-linked with the GA in the GO-GA solution. Furthermore, CGOPVA-50 consistently showed the well-concealed CPVA surface. The clear boundary between the GO-coated and exposed support region was observed at the edge of the membrane sample (Figure 4e). The cross-section of CGOPVA-50 revealed that the deposited GO-GA layer had a thickness ~67 nm, which could be an effective membrane barrier for solute rejections. 

### 3.2. Surface Properties of GOPVA and CGOPVA Membranes

**Figure 5:**
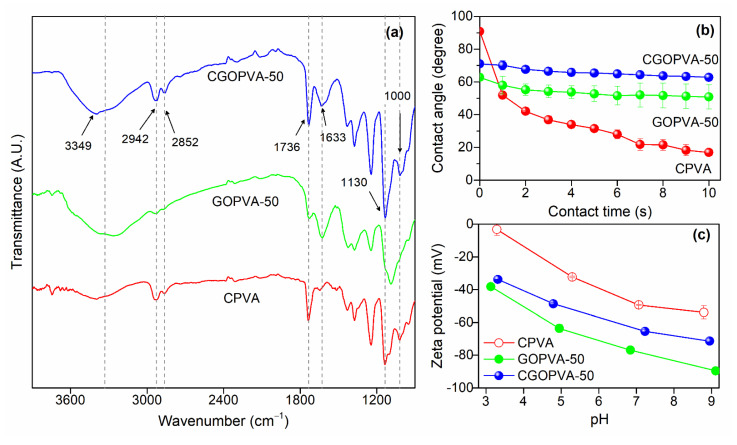
Characterization results of pure and GO-modified PVA nanofiber membranes: (**a**) FTIR; (**b**) contact angle; (**c**) zeta potential measurements.

The FTIR spectra (Figure 5a) of pure and modified CPVA nanofibers revealed the differences among samples due to the presence of the GO layer and GA cross-linking. All membranes exhibited –OH stretching at 3349 cm^−1^ due to the ubiquity of –OH groups in the PVA and GO layer, and the doublet of C–H stretching at 2942–2852 cm^−1^ from the alkyl components of PVA [42,44]. The C=O stretching at 1736 cm^−1^ in the CPVA support was due to the presence of unreacted GA as not all aldehydes reacted with the PVA polymer, which has a relatively low degree of hydrolysis (87~89% hydrolyzed) [52].

The deposition of the GO layer on the PVA nanofiber in GOPVA-50 and CGOPVA-50 membranes was reflected by the presence of C=C stretching at 1633 cm^−1^ from the sp^2^ carbons of GO [53]. The intensity of the C=O peak at 1736 cm^−1^ was lower in the GOPVA-50 sample as the deposition of uncross-linked GO partially concealed the CPVA support. On the other hand, this signal had higher intensity in the CGOPVA-50 membrane due to GA-GO cross-linking, suggesting the presence of unreacted aldehyde groups in its GA-GO layer [52]. Meanwhile, the appearance of C–O–C bonds at 1130 cm^−1^ and 1000 cm^−1^ in GA-cross-linked membranes signified acetal group formation. This confirmed successful GA cross-linking between PVA chains in CPVA, and the formation of GO-GA-PVA and GO-GA-GO linkages in CGOPVA-50 [54]. Moreover, as –OH groups were consumed during GA cross-linking, the same samples had reduced –OH signals relative to the uncross-linked analog, GOPVA-50. While the results from FTIR showed useful information on the modifications performed on PVA nanofibers, XPS analysis was conducted to acquire more insightful information on the deposited GO layer as discussed in the latter part of this section. The results from FTIR were supported by those from contact angle (Figure 5b) and zeta potential (Figure 5c) measurements. The contact angle of the uncoated CPVA support started at 90° (0 s), which is the highest initial contact angle among the tested samples due to the rougher surface of the exposed nanofibers [55]. It drastically declined to 51° after 1 s followed by a more gradual decrease until it reached 10° after 10 s. The decline in water contact angle of CPVA was mainly due to its highly porous structure, which allowed the water droplet to slowly percolate through its interstitial pores. Moreover, CPVA was still very hydrophilic even after GA cross-linking due to the abundant presence of residual –OH groups as confirmed by FTIR results (Figure 5a). Thus, the hydrophilic CPVA fibers likely contributed to the percolation of the water droplet. Meanwhile, both GOPVA-50 and CGOPVA-50 membranes exhibited lower initial contact angles than that of the CPVA support due to the smoother surface of the GO layer. The higher contact angle of CGOPVA-50 (70.98°) than GOPVA-50 (62.74°), which was not cross-linked with GA, agreed with the FTIR results. The loss of –OH groups due to acetalization with GA rendered CGOPVA-50 more hydrophobic than GOPVA-50. Nonetheless, the increase in contact angle was not significant, as GO also contained other oxygenous groups, including residual –OH groups that did not completely react with GA. The contact angle time profiles revealed generally steady values for both GOPVA-50 and CGOPVA-50 samples, which further confirmed the presence of a defect-free GO layer, which retarded the passage of water molecules through the membrane under static conditions. A similar trend was also observed in the zeta potential measurements of the membrane samples. Basically, all samples for CPVA, GOPVA-50, and CGOPVA-50 exhibited extremely negative surface charges at pH = 3–9, due to the abundant presence of deprotonated oxygen-containing hydrophilic functional groups such as hydroxyl and carboxylic groups [19,55]. It can be noted that the CGOPVA-50 showed a less negatively charged surface at all pH ranges than GOPVA-50. This can be attributed to the reduction in –OH groups on the GO layer after GA cross-linking, which agrees well with the results from contact angle measurements [19].

**Figure 6:**
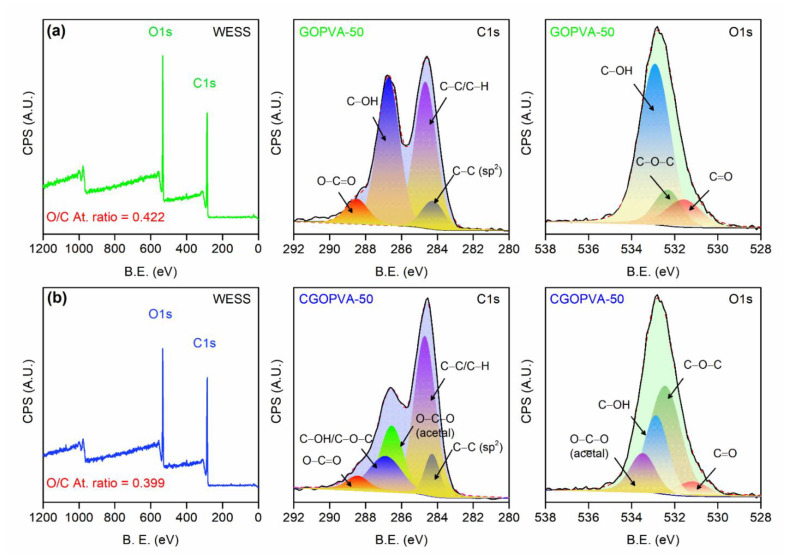
XPS spectra of (**a**) GOPVA-50 and (**b**) CGOPVA-50 samples.

The GOPVA-50 and CGOPVA-50 membranes were further examined via XPS (Figure 6) for a deeper understanding of the effect of GA cross-linking on the GO-selective layer. The wide energy scan spectra (WESS) of the samples were very similar, both containing C and O (Figure 6a,b). However, the comparison of their O/C atomic ratios showed the reduced value from 0.422 in GOPVA-50 to 0.399 in CGOPVA-50, which was consistent with the reduced –OH FTIR signals in the GA cross-linked membranes. Resolved peaks of the C1s spectra lines revealed the common peaks of the samples: sp^2^ C–C at 284.5 eV, C–C/C–H at 285.3 eV, C–OH at 286.85 eV, and O–C=O at 288.45 eV. However, the presence of O–C–O at 286.53 eV and C–O–C (overlapped with C–OH) in CGOPVA-50 further affirmed that GA successfully cross-linked with the GO nanosheets [56]. The oxygenous groups on GO were also seen in the O1s lines of both samples: O–C=O at 531.5 eV, C–O–C at 532.4 eV, and C–OH at 532.9 eV [53]. However, the CGOPVA-50 membrane showed a less intense C–OH peak concomitant with an increased C–O–C and the appearance of O–C–O (acetal) at 533.47 eV, which are all indicative of successful GA acetalization with GO and PVA. XPS results provided clearer evidence on the successful formation of a stable GO-selective layer on the PVA nanofibers through GA cross-linking.

### 3.3. NF Performance of GOPVA and CGOPVA Membranes

The measured PWP of the fabricated GO-coated composite membranes is summarized in Table 2. As shown, both uncross-linked (GOPVA) and GA cross-linked (CGOPVA) membranes exhibited PWP declines with GO filtration volume. This can be ascribed to the gradual increase in the resistance of the GO-selective layer as it thickened. This is because a higher amount of GO or GO-GA deposition also resulted in longer diffusion path lengths for the permeating water between the GO sheets (Figure 1), which generally reduced the PWP of the composite membranes. Furthermore, an increase in GO or GO-GA deposition resulted in more effective coverage of the CPVA surface, thereby reducing the defects on the membrane surface, which consequently alleviated nonselective convective water and solute permeation.

However, between the two types of membranes, the PWP of CGOPVA membranes was lower compared with the uncross-linked counterparts having the same amount of deposited GO. For example, GOPVA-10 had a higher PWP than CGOPVA-10 and so forth. Moreover, the PWP reduction in CGOPVA with increasing filtration volume was more obvious than that of the control GOPVA membranes. For instance, the PWP of GOPVA remained high even if 25 mL of GO was already deposited on the CPVA support. It was only reduced to 5.71 ± 1.87 L m^−2^ h^−1^ bar^−1^ when 50 mL of GO solution was filtered. In the case of the CGOPVA membrane, only 17.5 mL of GO-GA solution was needed to significantly reduce the PWP and reach a value <50 L m^−2^ h^−1^ bar^−1^ (CGOPVA-17.5 = 46.21 ± 3.12 L m^−2^ h^−1^ bar^−1^). It continuously declined to 11.94 ± 2.41 L m^−2^ h^−1^ bar^−1^ in CGOPVA-25 and 2.6 ± 1.08 L m^−2^ h^−1^ bar^−1^ in CGOPVA-50, which is within the PWP values of nanofiltration membranes [28,49]. These trends clearly show the benefit of GA cross-linking between GO nanosheets and GO with PVA support in creating a more stable and more selective GO layer. Without GA cross-linking, the GO nanosheets on the CPVA support would be unstable and would easily detach, creating more defects during operation, thereby resulting in high PWP values.

**Figure 7:**
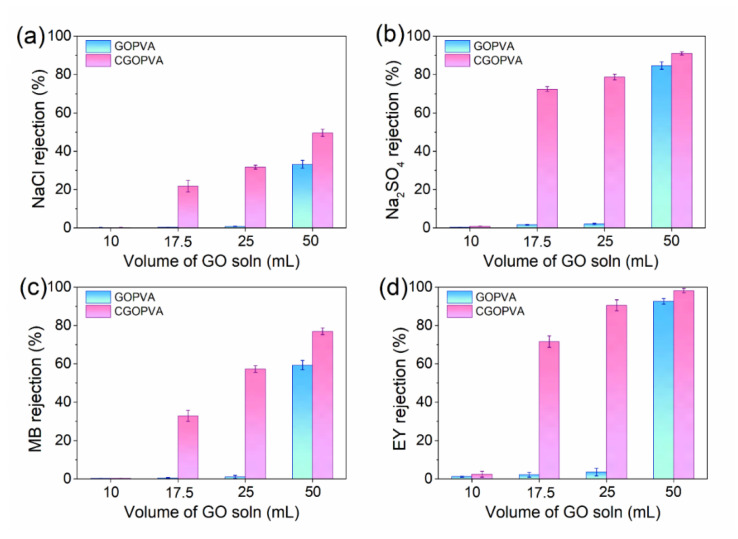
Rejection performances of GOPVA and CGOPVA membranes for (**a**) NaCl 20 mM, (**b**) Na_2_SO_4_ 20 mM, (**c**) MB 10 mg/L, and (**d**) EY 10 mg/L in terms of filtration volume of GO and GO-GA solutions (10, 17.5, 25, and 50 mL), applied pressure = 5 bar and cross-flow velocity = 8.3 cm^−1^.

The rejection performance of GOPVA and CGOPVA membranes at different filtration volumes of GO and GO-GA solutions, respectively, were systematically evaluated and compared using four different solutes such as NaCl, Na_2_SO_4_, MB, and EY feed solutions (Figure 7). From the figures, the rejections were improved with the increased volume of both GO and GO-GA coating solutions. However, in the case of uncross-linked GOPVA membranes, no effective rejection abilites were observed in any type of feed solution until GOPVA-25, because the GO did not fully cover the nanofiber surface, as evidenced by the FE-SEM image (Figure 4a), or the GO nanosheets were likely detached during operation. 

The rejection was also seen in GOPVA-50, as shown by the FE-SEM image (Figure 4b), where the CPVA was completely covered with a GO-selective layer. The control membrane exhibited a 33.12% rejection for NaCl, 84.66% for Na_2_SO_4_, 59.32% for MB, and 92.58% for EY. On the other hand, for GA cross-linked composite membranes, their rejection abilities were already observed in CGOPVA-17.5, which steadily increased until the highest values were achieved in CGOPVA-50. This trend further confirms that GA cross-linked CGOPVA membranes have better separation performances than their uncross-linked counterparts (GOPVA). The highest solute rejections were achieved in the CGOPVA-50 membrane, wherein it was able to reject EY at 98.12 ± 1.11%, Na_2_SO_4_ at 91.01 ± 0.87%, MB at 76.92 ± 1.74%, and NaCl at 49.62 ± 1.88%. The trend can be related to the size differences of the solutes as the molecular weight (MW) of EY (MW = 647.89 Da) was twice that of MB (MW = 319.85 Da), whereas NaCl was smallest and, hence, had the lowest rejection. Meanwhile, the rejection of Na_2_SO_4_ deviated from the trend as it was smaller than MB. This indicates that other mechanisms were likely involved aside from the ion sieving and the Donnan exclusion effects, which is addressed in the latter part of the discussion. 

**Figure 8:**
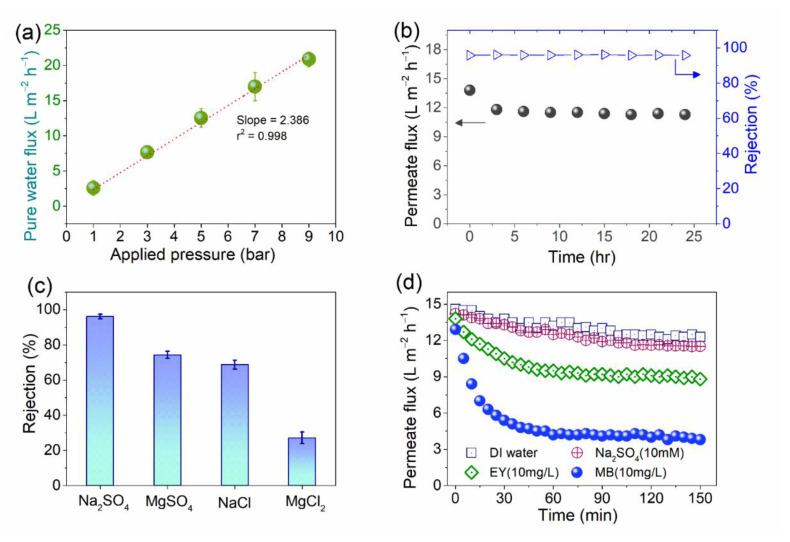
Membrane performances for CGOPVA-50: (**a**) PWP in terms of applied pressures (1–9 bar); (**b**) long-term membrane performance (feed solution: Na_2_SO_4_ 10 mM, applied pressure: 5 bar, and cross-flow velocity: 8.3 cm^−1^); (**c**) rejection performance under varied salts as a feed solution (10 mM of Na_2_SO_4_, MgSO_4_, NaCl, and MgCl_2_), applied pressure = 5 bar, and cross-flow velocity = 8.3 cm^−1^; (**d**) permeate flux trends under different feed solutions (applied pressure: 5 bar, and cross-flow velocity: 8.3 cm^−1^).

The membrane performances under varied conditions using the CGOPVA-50 membrane were further investigated (Figure 8). The PWP for CGOPVA-50 exhibited a linear increasing trend (*r*^2^ = 0.998) as the applied pressure was increased from 1 to 9 bars (Figure 8a). The slope from linear regression analysis estimates the increase in pure water flux of CGOPVA-50 per unit increase in applied pressure, which was around 2.386 L m^−2^ h^−1^ bar^−1^. This indicates that the cross-linked GO layer on the CPVA nanofiber support was mechanically stable and that the increase in pure water flux was steady with the applied pressure. Additionally, the stability of the CGOPVA-50 membrane was further evaluated for long-term membrane operation (24 h) at 5 bars with the cross-flow velocity of 8.3 cm s^−1^ using 10 mM of Na_2_SO_4_ as a feed solution (Figure 8b). Notably, there was a slight reduction in permeate flux from 13.8 L m^−2^ h^−1^ to 11.8 L m^−2^ h^−1^, which was observed within the first 3 h of operation. This could be mainly caused by membrane compaction under an applied pressure of 5 bar at the initial part of the run, but the permeate flux was maintained with a marginal decline onward for the rest of the operation. 

Meanwhile, rejection of Na_2_SO_4_ (10 mM) was consistently high (>95.8%) over the entire course of operation, confirming the good mechanical stability of the GO-selective layer. The rejection efficiencies of CGOPVA-50 at 5 bars for other salts (Figure 8c) showed that the composite membrane was most effective for Na_2_SO_4_, followed by MgSO_4_, then NaCl and, lastly, for MgCl_2_. This trend is typically observed with negatively charged nanofiltration membranes [47,55]. The CGOPVA-50 membrane had a highly negative charged surface, as confirmed by the zeta potential measurement (Figure 5c). The higher rejection efficiencies for sulfate (SO_4_^2−^)-containing salts such as Na_2_SO_4_ and MgSO_4_ than those for chloride (Cl^−^)-containing salts such as NaCl and MgCl_2_ can be due to the (1) molecular size sieving effect [49,57] and (2) Donnan exclusion effect, wherein the negatively charged divalent anion (SO_4_^2−^) is retained easier by the strong electrostatic repulsion with the negatively charged CGOPVA membrane surface [55].

The permeate flux of the CGOPVA-50 membrane in different types of feed solutions such as Na_2_SO_4_, MB, and EY were also investigated (Figure 8d). Compared with the PWP of CGOPVA-50, only a slight reduction in the permeate water flux was observed when 10 mM of Na_2_SO_4_ was used as a feed. The minor discrepancy can be due to the slight difference in the osmotic pressure at the feed solution. On the other hand, the permeate flux reductions for EY and MB were immediately observed at the initial stage of the operation, after 30 min. Unlike salts as solutes, dye molecules appeared to have more obvious fouling effects, which consequently reduced the water flux by absorbing or accumulating on the GO surface [34]. Interestingly, the water flux decline in the MB solution was more severe than that of EY, and this was due to the Donnan exclusion effect as 10 mg/L of EY solution (zeta potential: −48.7 ± 0.47 mV, analyzed by Zetasizer Nano ZS90) had a more negatively charged property compared with 10 mg/L of MB solution (zeta potential: −3.12 ± 1.26 mV, analyzed by Zetasizer Nano ZS90). The stronger electrostatic repulsion between the GO membrane surface and EY led to less fouling than the MB dye solution on the CGOPVA-50 membrane [24]. Overall filtration results showed the ability of CGOPVA-50 to reject certain ions and dyes, which makes the membrane applicable for wastewater treatment and water purification applications.

### 3.4. Physical Stability of GO-PVA Composite Membranes

**Figure 9:**
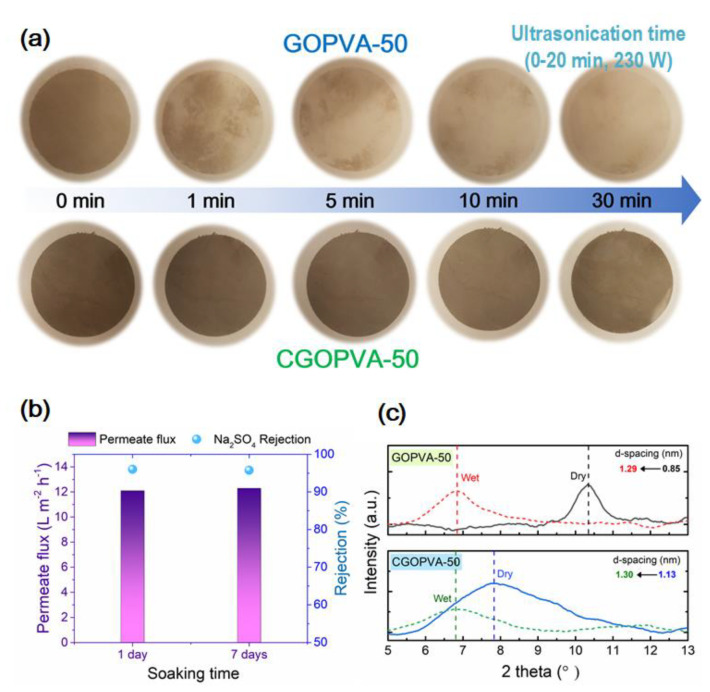
(**a**) Physical stability of GO-PVA composite membranes via ultrasonication method. (**b**) Membrane performance of CGOPVA-50 membrane as a function of soaking time in DI water (feed solution: Na_2_SO_4_ 10 mM, applied pressure: 5 bar, and cross-flow velocity: 8.3 cm^−1^). (**c**) XRD spectra and d-spacings of GOPVA-50 and CGOPVA-50 membranes at dry and wet states.

Optical images of sonicated membranes after a specified period of time are shown (Figure 9a) to observe the delamination of GO nanosheets from the PVA support. Without GA cross-linking, the GO nanosheets on GOPVA-50 were easily detached from the PVA substrate. A great portion of the GO layer was quickly exfoliated after 1 min and the entire GO layer almost disappeared after 30 min of sonication. On the other hand, the GO layer on the GA cross-linked CGOPVA-50 membrane was highly stable and remained intact even after 30 min. These results clearly demonstrate that GA cross-linking between GO sheets, as well as the GO layer with the PVA support, significantly improved the stability of the CGOPVA-50 membrane. 

The stability and reusability of the CGOPVA-50 membrane were further reflected by its consistent performance after soaking and storage in DI water (Figure 9b). The same CGOPVA-50 sample registered a consistent water flux and Na_2_SO_4_ rejection efficiency after one day and seven days of storage. These results (Figure 9a,b) and those from performance tests (Figure 7 and Figure 8) confirmed the benefit of GA cross-linking in improving the stability of CGOPVA-50, as the membrane was able to deliver good separation performance consistently for prolonged periods of time.

### 3.5. Influence of GA Cross-Linking on Membrane Performance

The effect of GA cross-linking on membrane performance was elucidated by comparing the interlayer distances (d-spacing) between the stacked GO nanosheets on GOPVA-50 and CGOPVA-50 membranes measured from their XRD patterns, Figure 9c. The swelling of the GO layer was assessed by comparing the d-spacings in the dry and wet state of each type of membrane.

In the dry-state, the d-spacing in GOPVA-50 was 0.85 nm, whereas that of CGOPVA-50 was 1.13 nm. The interlayer distance in CGOPVA-50 was enlarged as a result of the presence of GA linkages between the GO nanosheets. In the wet-state, the diffraction peak for GOPVA-50 considerably shifted to a lower 2θ value, which estimates a d-spacing of 1.29 nm. Similar behavior was observed for CGOPVA-50, which had a d-spacing of 1.30 nm. However, relative to their dry state, CGOPVA-50 experienced a lower swelling (% swelling = {value of d-spacing (wet)/value of d-spacing (dry)} × 100%) of 115.0% than that of the GOPVA-50 membrane (swelling ratio = 151.8%). This result indicates the important role of the GA cross-linker in reducing the swelling of the GO-selective layer [30,37,39,49].

Although the d-spacing of GOPVA-50 (1.29 nm) and CGOPVA-50 (1.30 nm) membranes were similar in the wet state, their NF performances (see Table 2 and Figure 7) were different. The presence of GA linkages in CGOPVA-50 likely added transport resistance to the GO-selective layer, hence its lower water permeance than that of GOPVA-50. On the other hand, the GA linkages in CGOPVA-50 might have introduced more tortuous paths between the GO nanosheets, hence its enhanced solute rejections than the GOPVA-50 membrane [29,38,58]. Although water permeance was reduced, GA cross-linking in CGOPVA-50 not only ensured the GO layer stability but also improved the solute rejections of the composite NF membrane.

## 4. Conclusions

Composite nanofiltration membranes were successfully synthesized by coating and cross-linking GO as a selective layer on a highly porous PVA nanofiber support with GA (CGOPVA). The efficient acetalization reaction of GA between GO nanosheets and GO with the PVA support was proven through a series of characterizations, which resulted in a mechanically stable composite membrane with effective separation performance. By adjusting the coating amount of the GO and GA cross-linker, the CGOPVA composite membrane was tuned until a defect-free GO layer was created on the support. The stability of the CGOPVA-50 composite membrane was confirmed by ultrasonication treatment, performance testing after prolonged soaking in DI water, and swelling measurement of the GO layer in the GA cross-linked membrane. The nanofiltration performances of the composite membranes were assessed in terms of solute selectivity and water permeance. At 5 bars, the most effective composite CGOPVA-50 membrane was able to reject 91.01 ± 0.87% for 20 mM of Na_2_SO_4_ and 49.62 ± 1.88% for 20 mM of NaCl. It was also capable of removing dye molecules such as EY at 98.12 ± 1.11% rejection and, to some extent, MB at 76.92 ± 1.74%. These results suggest that the implemented fabrication techniques could be promising in producing composite nanofiber-based NF membranes with an effective GO barrier for efficient solute rejections. 

## Figures and Tables

**Table 1:** Membrane properties of CPVA nanofiber substrate.

Sample	GA Cross-Linked PVA Substrate (CPVA)
Average fiber diameter (nm)	203.50 ± 26.17
Membrane thickness (µm)	67.52 ± 5.24
Porosity (%)	84.31 ± 1.97
PWP (L m^−2^ h^−1^ bar^−1^) ^(1)^	14,978 ± 894
Mean pore diameter (µm)	0.22
Maximum pore diameter (µm)	0.27

^(1)^ Applied pressure: 1 bar.

**Table 2:** PWP of GOPVA and CGOPVA membranes with different GO coating loadings.

Membranes	Volume of GO Filtration (mL)	GO Amount (µg cm^−2^)	PWP ^(1)^ (L m^2^ h bar^−1^)
GOPVA-10	10	4.91	9584 ± 749
GOPVA-17.5	17.5	8.60	4439 ± 398
GOPVA-25	25	12.28	897 ± 120
GOPVA-50	50	24.76	5.71 ± 1.87
CGOPVA-10	10	4.91	8436 ± 531
CGOPVA-17.5	17.5	8.60	46.21 ± 3.12
CGOPVA-25	25	12.28	11.94 ± 2.41
CGOPVA-50	50	24.76	2.6 ± 1.08

^(1)^ Applied pressure: 1 bar.

## Data Availability

No supporting data reported.
